# The Late Dr. Elisha Bartlett

**Published:** 1855-09

**Authors:** 


*The Late Dr. Elisha Bartlett.*

After a long illness, the issue of which has been but too plainly foreseen by all his friends, Dr. Elisha Bartlett has left us, regretted and honored throughout our whole land. His life has borne fruits to science and done good service to his fellow men in various spheres of duty. While we trust that it may find a faithful chronicler in some one of those who have been near him in its more active periods, it will not be out of place to devote a brief space in our pages to his memory. Hardly any American physician was more widely known to his countrymen, or more favorably considered abroad, where his writings had carried his name. His personal graces were known to a less extensive circle of admiring friends. and yet his image is familiar to very many who have received his kind attentions, or listened to his instructions or been connected with him in the administration of public duties.

To them it is easy to recall his ever welcome and gracious presence. On his expanded forehead no one could fail to trace the impress of a large and calm intelligence. In the most open and beaming smile none could help feeling the warmth of a heart which was a seat of all generous and kindly affections. When he spoke, his tone were of singular softness, his thoughts came in chosen words, scholarlike yet unpretending, often playful, always full of lively expression, giving the idea of one that could be dangerously keen in his judgments, had he not kept his fastidiousness to himself, and his charity to sheathe the weakness of others. In familiar intercourse—and the writer of these paragraphs was once under the same roof with him for some months—no one could be more companionable and winning in all his ways. The little trials of life he took kindly, and cheerily, turning into pleasantry the petty inconveniences which a less thoroughly good-natured man would have fretted over. A man so full of life will rarely be found so gentle and quiet in all his ways. A man who could be so satirical must have been very kind-hearted to let the sharp edge of his intellect be turned towards his neighbors' weaknesses so seldom. None was less disposed to put on airs in any company; he was rather too modest in coming out than too forward, though a silver-tongued speaker, to whom multitudes were always ready to listen whenever he was forced or beguiled to open his lips in public. I have been told that a distinguished foreign visiter who went through the whole length and breadth of the land, said that of all the many welcomes he received, from statesmen renowned as orators, from men whose profession is eloquence, not one was so impressive and felicitous as that which was spoken by Dr. Bartlett, then Mayor of Lowell, our brother in the Silent Profession, which he graced with these unwonted accomplishments. All these are now but pleasant memories; many eyes will grow dim as they are recalled, and many hearts beat warmly over them; when these eyes are darkened, and these hearts are stilled, the image just feebly traced will be like the shadow of yesterday.

But this is not all our friend left after him. It is hardly necessary here to refer to his public career as a magistrate. Yet this, though its results are less palpable to the public sense than those of scientific or literary labor, was in the highest degree honorable to his talent and integrity. Every man who has held manfully for any space any office in a system of government like ours, though the records of his doings may run through the spare fingers of history and sink into the sands, has done more and greater things than he can know—for no imagination can compass that future into which his courage and honesty shall enter as elements. To have left a high and cherished name after him in an officer so alien to his chosen pursuits and studies as the chief magistracy of a crowded city like Lowell, implies the possession of moral excellences as rare as the intellectual powers they accompanied. Had Dr. Bartlett fallen finally from his first love, and gone with his clear head and noble character and captivating oratory into the fatal passes of public life, it is paying our highest tribute to his virtues to say that he would certainly have been honored with the cross of high office, and at last with the crown of political martyrdom, the greatness of our civic wreaths in the time that are

The same qualities that fitted him for a public speaker, naturally given him signal success as a teacher. Had he possessed nothing but his remarkable clearness and eloquence of language and elocution, he could hardly have failed to find a popular welcome. Medical culture is often carried on among us by a light easy system of top-dressing. The rake is a more frequent instrument than the spade in the hands of many who are thought successful in rising the great harvest of students, the results of which are every March threshed and winnowed and garnered in our various schools. Among these, by all the qualities that give currency to the popular lecturer, by a manner at once impressive and pleasing, a lucid order which kept the attention and intelligence of the slowest hearer, and the attractions of a personal character always esteemed and beloved by students, he might have been prominent. With such he is not to be counted To accumulate without assimilating, to re-produce without enriching, to use rhetorical ornament to cover up the want of facts, to declaim instead of demonstrating, and to make all this pass current by an agreeable voice and easy confidence of manner; to do this is not difficult, and is both convenient and common. This was what Dr. Barlett did not do. His courteous and guarded language hardly betrayed his estimate of the class of mental operatives that live by such services, But he has lent the sharpest rebuke of the tribe to which they belong, in the sincerity and severe truth of his own writings.

As an author, Dr. Barlett is best known to the medical world by his Treatise on Fevers, and his Essay on Medical Philosophy. Few works not based upon long series of original observations have obtained or merited the consideration of the first of these treatises. He had the art of sifting authorities and getting at their essential meaning which belongs to the lawyer. He had the breadth and fairness of mind which enabled him weigh and decide on the masses of evidence before him; the same qualities that find their fullest expression in the voice of an enlightened judiciary. All might not accept his conclusions, but all could see that he was thoroughly faithful and honest, as well as able. Thus, his work on Fevers remains not only a most valuable monograph on these diseases, but a model for all who would produce a digest, as the lawyer call it, of whatever authentic knowledge is acquired upon any great medical question.

The Treatise on the Philosophy of Medicine is a work of wider aim and covering a ground open to more subtle controversy. It is the abstract expression of that phase of truth practically illustrated in the admirable works of Louis and his disciples. Clear and logical as everything he wrote, irresistible, if accepted as the development of truth in one direction, it has been reproached with throwing out of sight the higher qualities of imagination and invention in their legitimate applications to science. It is only fair, perhaps, to say that perfectly as it evolves its own conclusions, it would be less open to charges of omission if a chapter such as he himself might well have supplied, had been added upon the action of the inventive mind in the discovery of truth. The reader who will refer to the forcible and elegant lecture of Prof. Henry J. Bigelow, entitled "Fragment of Medical Science and Art," will find this point fully unfolded and illustrated. Not the less is Dr. Bartlett's essay of permanent excellence, because in the close logical pursuit of his chain of propositions, be has seemed to exclude principles which under another aspect his own imaginative mind would have been the first to recognize.

Everywhere through his writing prevails that easy flow of language, that felicity of expression, that florid warmth when occasion offers, which commonly marks the prose of those who are born poets. Yet few suspected him of giving utterance in rhythmical shape to his thoughts or feelings. It was only when his failing limbs could bear him no longer, as conscious existence slowly retreated from their palsied nerves, that he revealed himself freely in this trust and tenderest form of expression. We knew that he was dying by slow degrees, and we heard from him from time to time, or saw him, always serene and always hopeful while hope could have a place in his earthly future. His work was done, done nobly and gracefully, the work of an honest citizen, of a revered teacher, of a wise thinker. When to the friends he had loved, there came as a farewell gift not a last effort of the learning and wisdom they had been thought to expect from him, but a little book with a few songs in it, songs with his whole warm heart in them, they knew that his hour was come, and their tears fell fast as they read the loving thoughts that he had clothed in words of natural beauty and melody. The cluster of evening primroses had opened, and the night was close at hand.

No brief tribute like this can do more than show the feelings which its subject inspired in those who know him. He has left this earthly scene of his labors too early for friendship and for science, not for himself, ripe in every virtue and ready for wider spheres of knowledge; one of the "pure in heart," who look on the unveiled face of truth during their earthly pilgrimage, and who have the promise that they shall "see God" himself when they have reached its close.—O. W. H.—*Boston Med. & Sur. Journal.*

